# Servo Collision Detection Control System Based on Robot Dynamics

**DOI:** 10.3390/s25041131

**Published:** 2025-02-13

**Authors:** Qinjian Xiang, Chao Chen, Yadong Jiang

**Affiliations:** School of Optoelectronic Science and Engineering, University of Electronic Science and Technology of China, Chengdu 611731, China; 202212050802@std.uestc.edu.cn (Q.X.);

**Keywords:** industrial robot, collision detection, dynamics, current control, servo

## Abstract

Collision detection and inspection of industrial robots have become essential functions in modern industrial automation. Sensor-based detection methods are commonly employed in research to achieve collision detection, including high-precision force sensors, ultrasonic ranging sensors, electronic skins, and others. While collision detection using force sensors or electronic skin sensors offers very high accuracy, the inclusion of these sensors increases the overall cost. This article proposes a solution using dynamic modeling for collision detection. First, the theoretical torque generated by each axis of the industrial robot under different pose conditions is analyzed in real time. Then, the actual torque is calculated by sampling the motor current of each axis. By setting error margins and collision detection thresholds, collision detection can be achieved in a cost-effective manner without the need for additional sensors. Experiments were conducted to evaluate this dynamic modeling approach to collision detection. The findings indicated that the approach is efficacious and capable of identifying the impacts of diverse collision objects. However, compared to sensor-based detection methods, collision detection using dynamic modeling has the disadvantage of lower accuracy. Future research will concentrate on enhancing the calculation accuracy of the theoretical torque to enhance the sensitivity of collision detection.

## 1. Introduction

In recent years, as factory automation has advanced, the use of industrial robots has steadily increased. These robots have become an integral part of automated production lines, playing key roles in applications such as welding, material handling, sampling, assembly, and more [1]. As the functional requirements for industrial robots grow, collision detection has become an essential feature. Robots equipped with collision detection capabilities can protect both the robot body and control system, preventing significant damage in the occurrence of a collision [2,3].

Common collision detection methods for industrial robots include torque sensors, dual encoders, ultrasonic sensors, and other sensor-based approaches. Reference [4] introduces a novel distance evaluation methodology based on depth sensors for collision detection, representing a significant advancement in robotic collision detection techniques. References [5,6] employed a data-driven approach utilizing coupled dynamic data from manipulators with and without external contacts to design and train a multi-output neural network. This innovative framework enables the detection of unintended collisions and identification of impacted links solely through the manipulator’s intrinsic joint position and torque sensors, eliminating the need for additional external sensing devices. Reference [7] proposes a systematic methodology for quantifying the impact of current sensor drift on human–robot collision behavior, accompanied by the development of a data-driven compensation approach. In Reference [8], a novel methodology was introduced for collision detection by monitoring current variations in each joint of the robotic system. This approach leverages force sensor data mounted at the robot’s base, employing the principles of virtual displacement and force method to accurately determine the collision point location information at the impacted joint.

This article focuses on a collision detection method based on robot dynamics, which requires the development of an accurate robot dynamics model. By monitoring the motor currents of each axis, the torque output to each joint is calculated using motor parameters and the joint transmission ratio. Additionally, the position encoder provides feedback, which, when combined with the robot dynamics equations, helps determine the required driving torque for each joint in the current motion state. The calculated torque values are then compared to detect potential collisions. The dynamics of robots can be modeled using methods such as the Euler–Lagrange approach or the Newton–Euler equation, which analyze the relationship between joint motion and joint torque [9]. In addition, because robots use different loads in their actual work, it is necessary to set tool load parameters for the robot and incorporate them into dynamic calculations. In the process of modeling, it is necessary to accurately know the mechanical and motor parameters of industrial robots and calculate the speed and acceleration based on the feedback positions of each axis motor, as well as the theoretical torque, in order to ultimately determine the occurrence of collisions [10]. Since the theoretical torque is computed instantaneously according to the robot’s pose and the feedback torque is also sampled in real time, collisions can be detected promptly in various operating states [11].

In comparison with other methods, Reference [12] directly uses changes in motor current as an indicator of robot collisions. When a robot collides, its joints will suddenly be subjected to the torque produced by the collision force, which will change the load on the corresponding joints and cause a sudden change in the motor current. Therefore, the signal obtained by differentiating the current over time can be used as the collision detection signal. When the robot changes direction, it can also cause sudden changes in the robot’s joint torque, so the joint speed needs to be added to the detection signal. Through the experimental results of Reference [12], it was found that the method proposed in Reference [12] has the advantage of high accuracy. The detection threshold for joint 1 is only 0.5 NM, but the real-time performance is not high, with collision detection taking about 5–6 ms. Due to its ability to detect collisions through feedback torque, there is a possibility of misjudgment. Besides collisions, there are other situations that can cause an abnormal torque increase, such as motor brake damage, motor damage, or mechanical problems. This leads to misjudgment in this method. According to Reference [13], by utilizing CAD and setting up an impedance controller, collisions between robots can be effectively prevented. From the experimental results in Reference [13], the collision avoidance effect is relatively good, but the real-time performance is not high. The communication cycle between the controller and the PC reaches 150 ms, and it relies on CAD. Reference [14] demonstrates that using torque sensors provides a relatively simple, convenient, and effective method for detecting whether a robot has collided. The feedback torque based on torque sensors offers higher accuracy, but it still relies on the accuracy of the model. According to the experimental results in Reference [14], the torque sensor used exhibits high accuracy, with a threshold set at 0.8 Nm, and shows good performance in terms of real-time capability and false positive rate. However, the use of force sensors increases both structural and component costs.

Table 1 shows the methods currently used by some robot manufacturers to detect collision faults, which are mostly based on dual encoders and torque sensors.

## 2. Servo (Chengdu CRP Robot Technology Co., Ltd., Chengdu, China) System with Collision Detection Function

### 2.1. Hardware Platform

**Figure 1:**
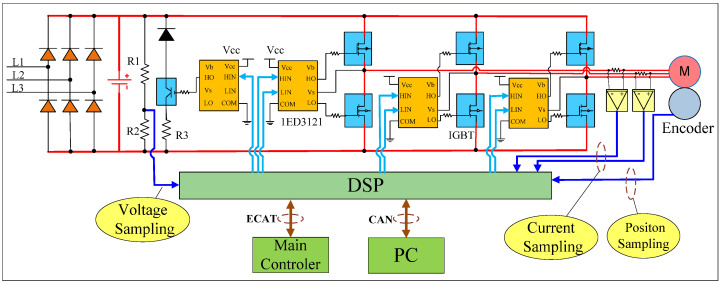
Topology diagram of servo hardware.

Figure 1 shows the complete servo hardware topology, with the servo driver as the core component for the robot’s collision detection system. Let us first discuss its hardware framework.

As the core component of the driving motor, servo drives play a crucial role in receiving commands from the main controller and controlling the motor in industrial robots. The entire hardware circuit includes a rectifier circuit, an inverter circuit, a digital signal processor (DSP) control circuit, and a sampling circuit. The rectifier circuit transforms three-phase alternating current into direct current. The power circuit’s primary function is to transform 220 VAC into various DC voltages, including VCC (5 V), 3.3 VDC, and ±15 V. These voltages primarily power the DSP and various other components like sampling chips, driver chips, and operational amplifier chips, as depicted in Figure 1. The inverter circuit includes a pulse-width modulation (PWM) drive circuit and an insulated gate bipolar transistor (IGBT). The PWM circuit is a key pulse-generating device for motor control. It produces a high-speed switching PWM signal that directly drives the motor, enabling it to rotate. This circuit is a fundamental part of the power system. The PWM driver chip model is 1ED3121, shown in orange in Figure 1. 1ED3121 is an IGBT gate driver chip designed for easy integration into reliable systems. The IGBT module, shown in blue in Figure 1, is responsible for switching the power to the motor.

The DSP control circuit includes the DSP core, along with analog and digital signal acquisition and processing circuits. As the core of servo control, DSP is the central processing unit of the entire servo system. Analog signals include motor current, bus voltage, and temperature, all of which are sampled by the DSP. After signal conditioning, they enter the DSP ADC channel for control and calculation. Digital signal processing circuits mainly include PWM wave generation, state quantity control, digital input signals, overcurrent protection signals, etc. [15]. Additionally, the DSP is responsible for communication with the main controller, including data processing through the Controller Area Network (CAN) for communication with the host computer. It also stores data and supports other functions. The DSP is connected to the main controller through communication methods such as Ethernet for Control Automation Technology (ECAT) communication, receives position and speed commands from the main controller, and feeds back information such as motor current, motor speed, and motor position sampled by the servo to the main controller. It form a closed-loop control loop [16].

### 2.2. Software Framework

**Figure 2:**
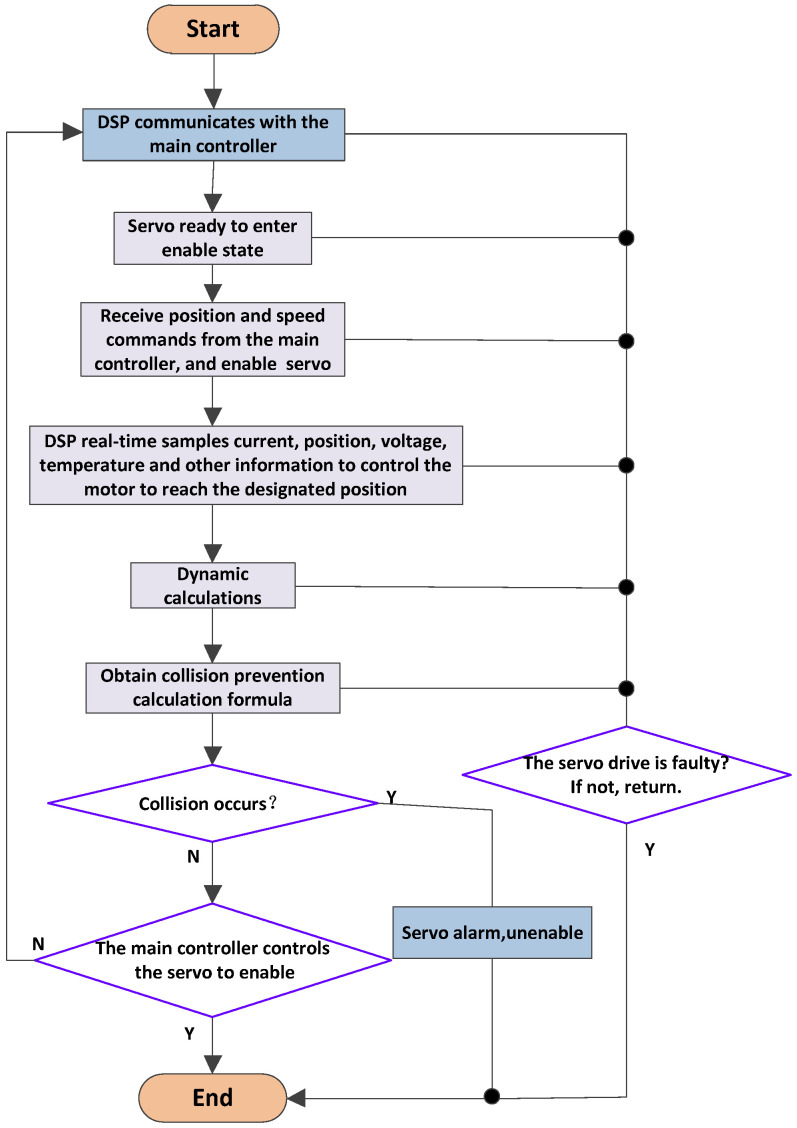
Software design flowchart.

As seen in Figure 2, the software control framework makes use of TI’s TMS320F28377D processor, a potent 32-bit floating-point MCU made for sophisticated closed-loop control applications, including servo motor control and industrial drives. This dual-core DSP operates at a maximum clock frequency of 200 MHz and provides 24 PWM peripheral interfaces, making it an ideal choice for controlling multiple motors. The chip can control up to four motors and offers a wide range of peripheral interfaces. In this paper, the PWM frequency is set to 10 kHz, with a control period and sampling period both of 100 µs. The clock frequency is 2.5 MHz, giving a clock cycle of 0.4 µs. It takes approximately 12.8 µs to sample data from one encoder and 25.6 µs to sample data from two encoders. The remaining time for other processes is 74.4 µs, which is sufficient. However, reducing the sampling time and increasing the sampling frequency could still offer benefits [17].

The entire software process begins with the communication module, which handles the sending and receiving of data. This module facilitates data exchange between the main controller and the servo system. It receives enable commands, position commands, speed commands, current commands, and stop commands from the main controller while also uploading information about the servo’s status, position, feedback speed, and motor current. Communication methods can vary, but stability in the communication cycle is critical. A typical communication cycle is 1–2 ms. The commonly used communication protocol is the ECAT communication protocol, which is an industrial Ethernet control protocol developed by the German company Beckhoff. It is very suitable for motion control. This article uses 28377D and requires the use of specialized ECAT slave data processing chips. Currently, specialized ECAT chips include ET1100 and LAN9252. If TI’s 28,388 is used, there is no need for a dedicated slave processing chip, as this chip already integrates the ECAT protocol’s processing functionality. In addition, it is also possible to use dual-port RAM instead of bus communication. The primary controller and servo may concurrently read from and write to the dual-port RAM, facilitating interaction between the main controller and servo data [18].

When the servo is ready, it waits for the main control command. Upon receiving the enable command from the main controller, the servo enters the enabled state and can receive position and speed commands. The main controller can also send only the position command without the speed command, if necessary [19]. While the main controller controls the servo’s position, the speed command, when provided, serves as a feedforward input to the speed control loop, potentially improving control performance. At the same time, the DSP continuously collects real-time data such as motor current, bus voltage, and temperature. The DSP then performs closed-loop control to adjust the motor and bring it to the desired position as specified by the main controller [20]. Additionally, the theoretical torque for each axis is computed according to the position, velocity, and acceleration data. The feedback torque is calculated from the motor current, and both values are compared to the collision threshold. If the threshold is exceeded, a collision warning is triggered, and the servo is activated to issue a collision alert. Otherwise, the motor control process continues as normal. Throughout the entire process, the servo will detect its own status in real time to see if any faults have occurred. If other faults occur, the servo will also be activated and enter a fault state, facilitating timely protection of the servo and robot [21].

### 2.3. Control Framework

**Figure 3:**
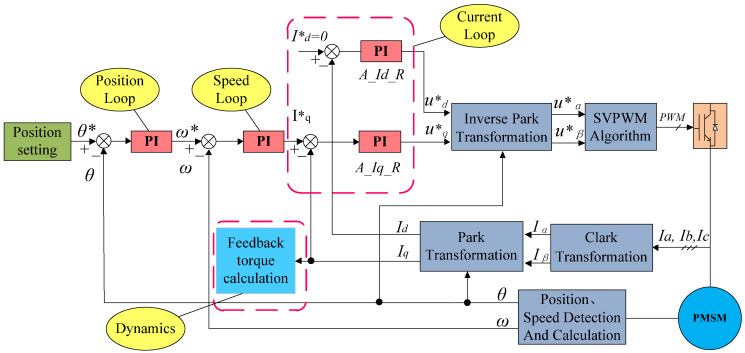
Servo control framework.

The entire control framework, as shown in Figure 3, consists of three servo protection control loops: the position control loop, the speed control loop, and the current control loop. The current control loop functions as the innermost loop, followed by the speed control loop as the middle loop and the position control loop as the outermost loop. After sampling the actual current of the motor with DSP, mathematical transformation is necessary for control. The motor current, which is a three-phase alternating current, is disadvantageous for control purposes [22].

In Figure 3, $\theta^{*}$ represents the position command transmitted from the main controller to the servo system, while $\omega^{*}$ denotes the output of the position loop, which simultaneously serves as the speed reference for the speed loop. The quantity ${I^{*}}_{q}$ corresponds to the output of the speed loop and functions as the current reference for the q-axis current loop, designated as *A_Iq_R.* Similarly, ${I^{*}}_{d}$ represents the current reference for the d-axis current loop, labeled as *A_Id_R*. For permanent magnet synchronous motors, ${I^{*}}_{d}$ is typically maintained at zero. The variable ${u^{*}}_{q}$ signifies the output of the q-axis current loop *A_Iq_R* and concurrently acts as one of the inputs to the Inverse Park Transformation module. Likewise, ${u^{*}}_{d}$ represents the output of the d-axis current loop *A_Id_R* and serves as another input to the Inverse Park Transformation module. Following this transformation, ${u^{*}}_{\alpha }$ and ${u^{*}}_{\beta }$ are generated as outputs of the Inverse Park Transformation module and subsequently serve as inputs to the Space Vector Pulse Width Modulation (SVPWM) Algorithm module.

To facilitate control, the three-phase alternating current must first be converted into two-phase orthogonal alternating current using mathematical formulas. This is followed by converting the two-phase alternating current into a two-phase direct current. This process involves the Clark transformation and the Park transformation. After applying these two transformations, the motor current is expressed as direct currents $I_{d}$ and $I_{q}$. They are the feedback of the d-axis current loop and the q-axis current loop, respectively [23]. The d-axis current is usually set to zero when controlling permanent magnet synchronous motors. The output of the q-axis current loop is determined by the speed loop, thereby forming two current control loops. In practical engineering applications, it is also necessary to filter the feedback and give the current separately. Here, $I_{q}$ also needs to calculate the feedback torque. As $I_{q}$ is the torque current of the motor, it must be transformed into each axis’s feedback torque. The conversion formula is as follows:

(1) $$T_{r}=I_{q}\times Q_{r}\times G\times R$$

In the Formula (1), $T_{r}$ is the feedback torque, $I_{q}$ is the torque current of the motor, $Q_{r}$ is the rated torque of the motor, *G* is the reduction ratio, and *R* is the direction of rotation, with values of 1 or −1. In addition to the current control loops, there is also the speed loop, which regulates the speed of the motor. The feedback speed is acquired using differentiating the encoder position. The speed setpoint for the speed loop is the position loop’s output. In engineering applications, filtering is also required for both the feedback speed and the given speed. The outermost loop is the position loop, which regulates the position of the motor. The position feedback is obtained from the encoder, while the position setpoint is provided by the main controller. Since the control cycle of the motor is usually shorter than the communication cycle, interpolation filtering is often used during the position-setting process. This completes the motor control framework as discussed in this paper [24].

**Figure 4:**
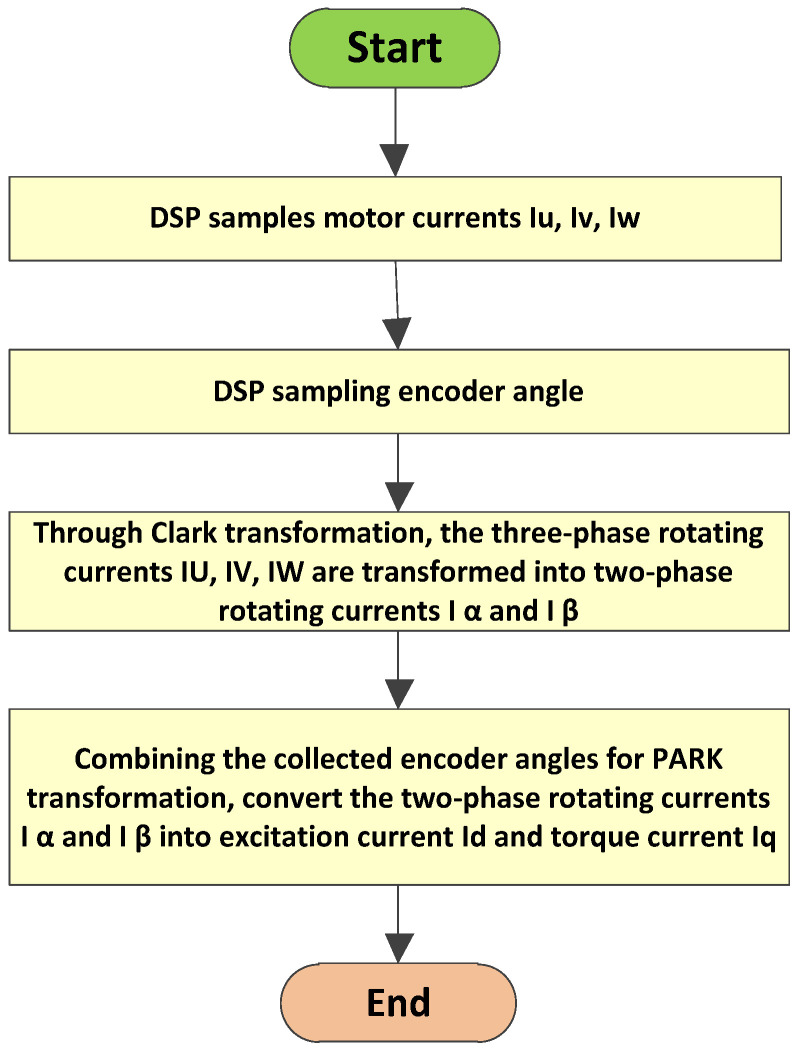
The calculation process of $I_{q}$.

How can we exactly acquire the torque current, $I_{q}$? To obtain the torque current, $I_{q}$, for closed-loop current control, a series of transformations must be performed on the sampled motor current. The process of calculating $I_{q}$ is shown in Figure 4.

DSP calculates the torque current, $I_{q}$, by collecting the actual motor current. The conversion from a three-phase alternating current (AC) to a two-phase alternating current is called Clark transformation, as shown in Formulas (2) and (3).

(2) $$I_{\alpha }=\frac{3}{2}I_{u}$$

(3) $$I_{\beta }=\frac{\sqrt{3}}{2}I_{v}-\frac{\sqrt{3}}{2}I_{w}$$

In the above equation, $I_{u}$ $I_{v}$ and $I_{w}$ represent the actual currents of the motor. $I_{\alpha }$ and $I_{\beta }$ are the currents after Clark transformation [25,26]. After applying the Clark transformation, the three-phase AC, exhibiting a phase difference of 120 degrees during motor rotation, is transformed into a two-phase AC with a phase difference of 90 degrees during motor rotation.

These two-phase orthogonal AC signals $I_{\alpha }$ and $I_{\beta }$ will be converted into DC currents $I_{q}$ and $I_{d}$ after undergoing Park transform.

The Park transformation formula is as follows:

(4) $$I_{d}=I_{\alpha }\cos\theta_{d}+I_{\beta }\sin\theta_{d}$$

(5) $$I_{q}={-I}_{\alpha }\sin\theta_{d}+I_{\beta }\cos\theta_{d}\;$$

A is the electrical angle, and its calculation formula is as follows:

(6) $$\theta_{d}=(\theta +\Delta \theta )\times P$$

*Θ* is the angle of the encoder, and Δθ is the electrical angle offset, which could be 0°, 90°, 180°, or other angles. Typically, these data are provided by the motor manufacturer. If there are no electrical angle offset data, the $I_{d}$ current loop can be used to control and determine the electrical angle offset, Δθ. However, since this is not the focus of this paper, the specific methods will not be described in detail. P is the number of motor poles.

By using Formulas (2)–(6) and following the steps shown in Figure 4, the torque current, $I_{q}$, can be obtained.

## 3. Collision Detection Dynamic Modeling

### 3.1. Kinematic Modeling

Kinematics and dynamics analysis are fundamental for the study of industrial robots. Kinematic analysis refers to the static analysis of robots, which delineates the correlation between a robot’s joint angles and the posture of its end effector. Kinematics is divided into forward kinematics and inverse kinematics. The inverse solution determines the joint angles in accordance with the desired end effector pose, whereas the forward solution determines the robot’s end effector position based on its joint angles. This is the foundation of robot trajectory planning. This article focuses on a robot with a 50 kg load. Based on its structure, its coordinates are initially established using the six-axis coordinate system (6) from the base coordinate system (0). Then, modified D-H parameters will be established based on the coordinates. The D-H parameter method is a coordinate transformation approach based on homogeneous transformation matrices, originally proposed in 1955 by Denavit and Hartenberg. Using a 4 × 4 homogeneous transformation matrix, this technique explains the spatial link between adjacent robot linkages and the robot’s kinematics through homogeneous transformations. The D-H approach is the name of this modeling technique. The D-H approach is still frequently applied today and is crucial to the advancement of robotics [27,28].

The regular D-H approach and the modified D-H method are now the two main categories of D-H modeling techniques. The primary distinction between these two approaches is that the connecting rod coordinate system {i} is positioned differently. The coordinate system {i} is established on the axis of the i + 1 joint by the normal D-H approach, whereas the coordinate system {i} is established on the axis of the i joint by the modified D-H method. Obviously, this alteration also results in variations in the D-H matrix and D-H parameters. The standard D-H method, proposed by Denavit and Hartenberg, is primarily designed for serial mechanisms. However, it can face challenges when modeling tree structures (such as when two branches are connected at the end of a linkage) or closed-chain structures. In contrast, the improved D-H approach avoids these problems; it is more adaptable and suitable for a wider range of robotic systems [29].

**Figure 5:**
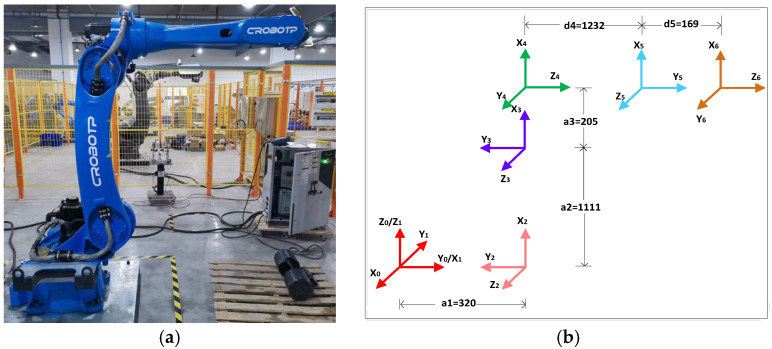
(**a**) Industrial robots are used in actual testing. (**b**) A six-axis coordinate system based on actual industrial robots.

Determining the modified D-H parameters is essential to building the kinematic model of industrial robots. To establish these parameters, it is essential to first define a coordinate system for the six axes of the industrial robot. The six-axis coordinate system, along with parameters established for a 50 kg load robot, is displayed in Figure 5b. Figure 5a illustrates the actual industrial robot used in the project [30].

Once the modified D-H coordinate system is defined, the modified D-H parameter table can be filled in based on the actual robot’s link length and link offset data. In the D-H parameter table, $a_{i-1}$ is the length of connecting link *i* − 1, defined as the length of the common normal between the axis of joint *i* − 1. Essentially, it is the shortest spatial distance between the axes of two consecutive joints. $\alpha_{i-1}$ is the connecting link *i* − 1 torsion angle, which is the angle pointing from axis i − 1 to axis i between the axis of joint *i* − 1 and the axis of joint *i*. $d_{i}$ is the offset of link i relative to link *i* − 1, defined as the distance between the two common normal lines, $a_{i}$ and $a_{i-1}$, on joint *i*. $\theta_{i}$ is the joint angle, which is the angle at which connecting link *i* rotates around axis *i* in relation to connecting rod *i* − 1. It is measured around joint axis i and points from $a_{i-1}$ to $a_{i}$.

After the updated D-H coordinate system has been established and the changed D-H parameters for the neighboring links have been established, the relationship between the end effector pose, joint angles (*θ*), and modified D-H parameters can be obtained by the use of the modified D-H transformation matrix. From the knowledge of robotics, the coordinate system {*i*} transformation matrix formula with respect to coordinate system {*i* − 1} is as follows:

(7) Tii−1=Rot(X,  αi−1)Trans(X,  ai−1)Rot(Z,θi)Trans(Z, di)=cosθisinθicosαi−1sinθisinαi−10   −sinθicosθicosαi−1cosθisinαi−10   0−sinαi−1cosαi−10    ai−1−sinαi−1dicosαi−1di1

In Formula (7), Tii−1 represents the pose transformation matrix from joint i to joint i − 1, $Rot(X,{\;\alpha }_{i-1})$ represents a rotation of $\alpha_{i-1}$ degrees around the *X*-axis, $Trans(X,{\;\alpha }_{i-1})$ represents a translation of ${\;a}_{i-1}$ meters along the *X*-axis, $Rot(Z,\theta_{i})$ represents a rotation of ${\;\theta }_{i-1}$ degrees around the *Z*-axis, and $Trans(Z,\;d_{i})$ represents a translation of $d_{i}$ meters along the *Z*-axis [31].

For an industrial robot with six joints, when the updated D-H coordinate system has been established and the adjusted D-H parameters between neighboring coordinate systems have been established, six modified D-H matrices can be calculated. By sequentially multiplying these matrices, the robot’s kinematic model is obtained. The kinematic model of a six-joint industrial robot is as follows:

(8) T60=T10T21T32T43T54T65=T(θ1)10T(θ2)21T(θ3)32T(θ4)43T(θ5)54T65(θ1)

T60 represents the pose transformation matrix from joint 6 to the base coordinate system, T10 represents the pose transformation matrix from joint 1 to base coordinate system (0), and so on. According to Formula (8) and the improved D-H parameter in Table 2, it can be calculated that T(θ1)10, T(θ2)21, T(θ3)32, T(θ4)43, T(θ5)54, T65(θ6). Then, how the robot’s final position is represented in the base coordinate system 0 is determined.

(9) T(θ1)10=cosθ1sinθ100   −sinθ1cosθ100   0010   0001

(10) T(θ2)21=cosθ2sinθ200   −sinθ2cosθ200   0010   0001

(11) T(θ3)32=cosθ3sinθ300   −sinθ30cosθ30   0−100   0.32001

(12) T(θ4)43=cosθ4sinθ400   −sinθ4cosθ400   0010   1.111001

(13) T(θ5)54=cosθ50sinθ50   −sinθ50cosθ50   0−100   0.205−1.23201

(14) T(θ2)21=cosθ2sinθ200   −sinθ2cosθ200   0010   0001

Thus, the equation between T60 and D-H parameters can be calculated.

(15) T60=Tθ110Tθ221Tθ332Tθ443Tθ554T65θ1=nxnynz0   oxoyoz0   axayaz0   pxpypz1=  R60(θ)p60(θ)01 

In Equation (15), $\left[\begin{array}{ccc} n_{x} & o_{x} & a_{x}\\ n_{y} & o_{y} & a_{y}\\ n_{z} & o_{z} & a_{z} \end{array}\right]$ is the pose coordinate of the end effector of the robot, represented by R60(θ). $\left\lfloor \begin{array}{c} p_{x}\\ p_{y}\\ p_{z} \end{array}\right\rfloor$ is the position coordinate of the robot’s end effector, represented by ${\;p}_{6}^{0}(\theta )$.

### 3.2. Dynamics Modeling

The dynamic collision detection function studied in this article is integrated into the servo system. Since the main task of the servo is to drive the motor, the collision detection function is considered a functional module of the servo. This servo-based collision detection is rooted in robot dynamics, meaning that dynamic modeling must be performed first [32].

Dynamic analysis explains the connection between industrial robots’ joint motion and joint torque. The required torque can be computed according to the current motion state of the robot’s joints, which is crucial for dynamic collision detection. For industrial robot control, kinematic and dynamic analysis facilitates the control of robot position and force. For collision detection of industrial robots, kinematic and dynamic analysis can easily obtain information such as the position, velocity, and torque of each joint of the robot in a certain state, which facilitates the judgment of the working state of the industrial robot and combined with actual torque feedback, determines the occurrence of collision events. Therefore, dynamic analysis forms the foundation for effective dynamic collision detection.

Methods for dynamic analysis of industrial robots include the Newton–Euler method, Kane method, and Euler–Lagrange method [33,34]. The forward and inverse solutions of robot kinematics reflect the relationship between robot joint angles and robot end effector positions, while the Jacobi matrix reflects the relationship between robot joint rotation speed and end effector linear and angular velocities. Therefore, the Jacobian matrix is a critical component in both the analysis and control of robot motion, playing a key role in deriving dynamic equations and collision detection algorithms.

#### 3.2.1. Jacobi Matrix

The inverse kinematics solution at the position level can be divided into geometric and algebraic methods. The final solution is an explanatory formula for the robot’s joint position variables. These methods have different specific calculation processes for different robots. Moreover, robots must meet the condition that there exists an analytical solution for the robot’s inverse kinematics. However, not every robot’s inverse kinematics can be solved analytically. So alternative methods are required for these cases. The Jacobi method at the speed level has the same inverse kinematics calculation process for different robots and does not require analytical solutions for the inverse kinematics of the robot. It is a universal inverse kinematics solution method. Compared to the position-level solution method, the disadvantage of the Jacobi method is its higher computational cost, which includes the calculation of the Jacobi matrix, leading to slower execution. However, its advantage lies in its broader applicability. The performance requirements for the Microcontroller Unit (MCU) are higher, so MCU chips with faster clock frequencies are necessary [35,36].

The Jacobi matrix represents the relationship between the rotational speed of the robot’s joints and the linear and angular velocities at the end effector of the robot. Various methods exist for calculating the Jacobi matrix, primarily including the method of differentiating the pose equation, the recursive link speed method, and the vector multiplication and differential transformation method. This article focuses on the vector multiplication method, which constructs the Jacobi matrix through link velocity analysis [37].

Industrial robots use rotary joints, so this article introduces the Jacobi matrix of rotary joints. For the rotating joint, i, the linear velocity and angular velocity are as follows:

(16) $$\left[\begin{array}{c} v_{i}\\ \omega_{i} \end{array}\right]=\left[\begin{array}{c} {z_{i}\;\times }^{i}\;p_{n}^{0}\\ z_{i} \end{array}\right]{\dot{q}}_{i}$$

In Equation (16), $z_{i}$ is the unit vector of the robot’s *i*-joint in the z-direction, which is the third column of the T6i matrix. ${\dot{q}}_{i}$ is the derivative of the *i*-joint angle, and *^i^*$p_{n}^{0}$ is the position vector of the robot’s end effector origin relative to the coordinate system {*i*} in the base coordinate system {0}. Therefore, the Jacobi matrix of joint *i* is:

(17) $$J_{i}=\left[\begin{array}{c} J_{vi}\\ J_{\omega i} \end{array}\right]=\left[\begin{array}{c} {z_{i}\;\times }^{i}\;p_{n}^{0}\\ z_{i} \end{array}\right]$$

(18) pn0=Rii0pn

where Ri0 represents the attitude transformation matrix between the joint coordinate system {*i*} and the base coordinate system {0}, and *^i^*$p_{n}$ is the position vector of the robot’s end-effector in the coordinate system {*i*}.

For a six-joint robot, the Jacobi matrix constructed by vector cross multiplication is as follows:

(19) $$J\left(q\right)=\left[\begin{array}{c} {z_{1}\;\times }^{1}\;p_{6}^{0}\\ z_{1} \end{array}\;\;\begin{array}{c} {z_{2}\;\times }^{2}\;p_{6}^{0}\\ z_{2} \end{array}\;\;\;\begin{array}{c} \ldots \\ \ldots \end{array}\;\;\;\begin{array}{c} {z_{6}\;\times }^{6}\;p_{6}^{0}\\ z_{6} \end{array}\right]\;=[J_{1}\;\;J_{2}\;\;J_{3}\;\;J_{4\;\;}J_{5}\;\;J_{6}]$$

Therefore, the linear velocity and angular velocity at the end of the robot can be expressed as follows:

(20) $$\left[\begin{array}{c} v_{6}\\ \omega_{6} \end{array}\right]=\left[\begin{array}{c} {z_{1}\;\times }^{1}\;p_{6}^{0}\\ z_{1} \end{array}\;\;\begin{array}{c} {z_{2}\;\times }^{2}\;p_{6}^{0}\\ z_{2} \end{array}\;\;\;\begin{array}{c} \ldots \\ \ldots \end{array}\;\;\;\begin{array}{c} {z_{6}\;\times }^{6}\;p_{6}^{0}\\ z_{6} \end{array}\right]\left[\begin{array}{c} \begin{array}{c} {\dot{q}}_{1}\\ {\dot{q}}_{2}\\ {\dot{q}}_{3} \end{array}\\ {\dot{q}}_{4}\\ {\dot{q}}_{5}\\ {\dot{q}}_{6} \end{array}\right]$$

For a six-degree-of-freedom industrial robot, the following relationship holds:

(21) $${\left[{\dot{q}}_{1}\;{\dot{q}}_{2}\;{\dot{q}}_{3}\;{\dot{q}}_{4}\;{\dot{q}}_{5}\;{\dot{q}}_{6}\right]}^{T}={\left[{\dot{\theta }}_{1}\;{\dot{\theta }}_{2}\;{\dot{\theta }}_{3}\;{\dot{\theta }}_{4}\;{\dot{\theta }}_{5}\;{\dot{\theta }}_{6}\right]}^{T}$$

Therefore, Equation (20) can be written as follows:

(22) $$\left[\begin{array}{c} v_{6}\\ \omega_{6} \end{array}\right]=\left[J_{1}\;\;J_{2}\;\;J_{3}\;\;J_{4\;\;}J_{5}\;\;J_{6}\right]\left[\begin{array}{c} \begin{array}{c} {\dot{\theta }}_{1}\\ {\dot{\theta }}_{2}\\ {\dot{\theta }}_{3} \end{array}\\ {\dot{\theta }}_{4}\\ {\dot{\theta }}_{5}\\ {\dot{\theta }}_{6} \end{array}\right]$$

#### 3.2.2. Robot Body Dynamics Modeling

The key to the dynamic analysis of a robot’s body is to establish the dynamic equations for industrial robots and to solve for the various parameters in these equations. This article uses the Euler–Lagrange method to derive the dynamic equations. For a single degree of freedom system with complete constraints, using the Euler–Lagrange approach and beginning with the virtual work concept, the Euler–Lagrange equation can be derived [38].

(23) $$\frac{d}{dt}\frac{\partial L}{\partial \dot{y}}-\frac{\partial L}{\partial y}=f$$

In Equation (23), *y* represents the generalized coordinate, and $\dot{y}$ is the generalized velocity. The symbol *L* is the Lagrange operator, which represents the difference between the kinetic energy and potential energy of the system: *L = K − P*, where *K* is kinetic energy and *P* is potential energy; in Equation (23), f represents the generalized force acting on the system, excluding gravity. Applying Equation (23) to industrial robot systems, the Euler–Lagrange equation of the robot can be obtained:

(24) $$\frac{d}{dt}\frac{\partial L}{\partial \dot{\theta_{i}}}-\frac{\partial L}{\partial \theta }=\tau_{i}$$

In Equation (24), $\theta_{i}$ is a generalized coordinate representing the angle of the robot joint, ${\dot{\theta }}_{i}$ is the acceleration of robot joint *i*, and $\tau_{i}$ represents the torque applied to the connecting rod of the corresponding joint.

Next, we calculate the Lagrange operator, *L*, starting with the kinetic energy. Translational and rotational kinetic energy make up the two components of the robot’s joint kinetic energy. For simplicity, the mass of the connecting rod is concentrated at the mass position’s center. Therefore, the kinetic energy of the link is as follows:

(25) $$K=\frac{1}{2}m_{i}v_{i}^{T}v_{i}+\frac{1}{2}\omega_{i}^{T}I_{i}^{0}\omega_{i}$$

In Equation (25), $m_{i}$ represents the mass of the connecting link, *i*; $v_{i}$ and $\omega_{i}$, respectively, represent the linear velocity and angular velocity of the robot link, *i*; and $I_{i}^{0}$ represents the inertia tensor of the link, *i*, relative to the base coordinate system {0}. The position of the joint, *i*, relative to the base coordinate system is generally uncertain. For ease of calculation, the inertia tensor, $I_{i}$, of the link, *i*, relative to the joint coordinate system can be calculated first and then transformed into the inertia tensor, $I_{i}^{0}$, of the link, *i*, relative to the base coordinate system. The conversion formula is as follows:

(26) Ii0=Ri0IiRi0T

In Equation (26), Ri0 represents the rotation matrix between the joint coordinate system, *i*, and the base coordinate system. The inertia tensor, $I_{i}$, is a constant matrix that is independent of joint motion.

(27) $$I_{i}=\left[\begin{array}{ccc} I_{XX} & I_{XY} & I_{XZ}\\ I_{YX} & I_{YY} & I_{YZ}\\ I_{ZX} & I_{ZY} & I_{ZZ} \end{array}\right]$$

In Equation (27),

(28) $$\left\{\begin{array}{c} I_{XX}=∭(y^{2}+z^{2})\rho (x,y,z)dxdydz\\ I_{YY}=∭(x^{2}+z^{2})\rho (x,y,z)dxdydz\\ I_{ZZ}=∭(x^{2}+y^{2})\rho (x,y,z)dxdydz\\ I_{XY}=I_{YX}=-∭xy\rho (x,y,z)dxdydz\\ I_{XZ}=I_{ZX}=-∭xz\rho (x,y,z)dxdydz\\ I_{YZ}=I_{ZY}=-∭yz\rho (x,y,z)dxdydz \end{array}\right.$$

ρ(x,y,z) is the density of the link, and *x*, *y*, and *z* are the coordinates of the link. Due to the complexity of directly calculating the inertia tensor, $I_{i}$, this paper uses 3D modeling and Solidworks software (2023 version) to analyze and calculate the inertia tensor of the link, i, relative to the joint coordinate system [39].

Because $v_{i}=J_{v_{i}}(\theta )\dot{\theta }$, $\omega_{i}=J_{\omega_{i}}(\theta )\dot{\theta }$

(29) $$K=\frac{1}{2}m_{i}v_{i}^{T}v_{i}+\frac{1}{2}\omega_{i}^{T}I_{i}^{0}\omega_{i}\;=\frac{1}{2}{\dot{\theta }}^{T}\left\{\sum_{i=1}^{6}\left[m_{i}J_{v_{i}}{\left(\theta \right)}^{T}J_{v_{i}}\left(\theta \right)+m_{i}J_{\omega_{i}}{\left(\theta \right)}^{T}R_{i}^{0}I_{i}R_{i}^{0T}J_{\omega_{i}}\left(\theta \right)\right]\right\}\dot{\theta }=\frac{1}{2}{\dot{\theta }}^{T}D\left(\theta \right)\dot{\theta }$$

In Equation (29),

(30) $$D\left(\theta \right)=\sum_{i=1}^{6}\left[m_{i}J_{v_{i}}{\left(\theta \right)}^{T}J_{v_{i}}\left(\theta \right)+m_{i}J_{\omega_{i}}{\left(\theta \right)}^{T}R_{i}^{0}I_{i}R_{i}^{0T}J_{\omega_{i}}\left(\theta \right)\right]$$

$D\left(\theta \right)$ is the inertia matrix of the robot, which is a symmetric matrix and positive definite.

Let

(31) $$\left[d_{ij}\right]=\left[m_{i}J_{v_{i}}{\left(\theta \right)}^{T}J_{v_{i}}\left(\theta \right)+m_{i}J_{\omega_{i}}{\left(\theta \right)}^{T}R_{i}^{0}I_{i}R_{i}^{0T}J_{\omega_{i}}\left(\theta \right)\right]$$

Equation (29) can be written as follows:

(32) $$K=\frac{1}{2}\sum_{i,j}^{6}d_{ij}\left(\theta \right){\dot{\theta }}_{i}\;{\dot{\theta }}_{j}$$

*d*_*ij*_ is an element in the inertia matrix, $D\left(\theta \right)$, and the Lagrange operator can be transformed into the following:

(33) $$L=K-P=\frac{1}{2}\sum_{i,j}^{6}d_{ij}\left(\theta \right){\dot{\theta }}_{i}\;{\dot{\theta }}_{j}-P(\theta )$$

By substituting Equation (32) into the Lagrange Equation (24), we can obtain the following:

(34) $$\sum_{j}d_{ij}{\ddot{\theta }}_{j}+\frac{1}{2}\sum_{i,j}\left[\frac{{\partial d}_{kj}}{{\partial \theta }_{i}}+\frac{{\partial d}_{kj}}{{\partial \theta }_{i}}-\frac{{\partial d}_{ij}}{{\partial \theta }_{k}}\right]{\dot{\theta }}_{i}\;{\dot{\theta }}_{j}+\frac{\partial P}{{\partial \theta }_{k}}=\tau_{k}\;$$

In Equation (34), the servo can calculate $d_{ij}$ by setting various dynamic parameters and sampling the encoder position in real time, while $\theta_{i}$ is the encoder position, which can be sampled in real time. Using these data, the torque for each axis can be calculated using the above equation [40].

The potential energy, P, of the robot is considered next. Since the industrial robot used in this paper is a rigid robot, the potential energy only needs to account for gravitational potential energy. Assuming the mass of the joint is centered at the mass center the gravitational potential energy of the link, *i*, is given by the following:

(35) $$P_{i}=m_{i}g^{T}r_{ci}\;$$

In Equation (35), *g* is the gravity vector in the base coordinate system {0}; $r_{ci}$ represents the coordinates of the centroid of the joint, *i*, in the base coordinate system. The total potential energy of the robot is as follows:

(36) $$P=\sum_{i=1}^{6}P_{i}=\sum_{i=1}^{6}m_{i}g^{T}r_{ci}$$

Therefore, Equation (34) can be written as:

(37) $$\sum_{j=1}^{6}d_{jk}{\ddot{\theta }}_{j}+\frac{1}{2}\sum_{i,j}\left[\frac{{\partial d}_{kj}}{{\partial \theta }_{i}}+\frac{{\partial d}_{kj}}{{\partial \theta }_{i}}-\frac{{\partial d}_{ij}}{{\partial \theta }_{k}}\right]{\dot{\theta }}_{i}{\dot{\theta }}_{j}+\sum_{i=1}^{6}m_{i}g^{T}\frac{\partial r_{ci}}{{\partial \theta }_{k}}=\tau_{i}$$

The torque of axes 1–6 can be calculated in real time using Equation (37). In practical engineering applications, due to the servo’s sampling rate for encoder positions and the speed at which calculations are performed, the torque calculation algorithm is often implemented directly within the servo to enhance computation speed.

Because the robot tools influence the six-axis dynamic model, it is necessary to model each tool individually to determine its centroid position, mass, and calculate the moment of inertia. As a result, even for the same robot, variations in the tool load will lead to differences in the center of mass and mass distribution across the six axes. The tool’s characteristics significantly affect the calculated torque and the feedback torque of each axis of the robot [41]. Table 3 displays the values needed for dynamic collision detection.

The parameters required for dynamic collision detection are shown in Table 3, including joint mass, centroid position, moment of inertia, motor rated current, motor rated torque, and other parameters. The inertia moment parameter is calculated through mechanical software (Solidworks 2023 version).

The dynamic modeling approach indeed increases computational overhead but does not affect control performance. In this study, the highest priority is given to motor current sampling, encoder sampling, and motor control, followed by fault protection mechanisms such as overcurrent detection. The third priority is the torque calculation for dynamic modeling. The system utilizes a DSP chip with a 200 MHz clock frequency, and the entire dynamics-based collision detection computation takes approximately 275 microseconds, which satisfies real-time requirements. Employing a higher-frequency chip would further reduce computation time and enhance real-time performance.

In reality, robotic systems exhibit nonlinear characteristics, such as those introduced by inverter nonlinearities and dead-time effects. These nonlinearities can be effectively mitigated through the implementation of compensation strategies.

Additionally, friction compensation plays a crucial role in enhancing the accuracy of calculated torque. Friction torque is typically obtained through experimental measurements, and motor manufacturers often provide these data. However, it is common practice to experimentally verify the friction torque parameters supplied by manufacturers. The parameter table has been updated to include both Coulomb friction and the coefficient of kinetic friction. In practical calculations, if the coefficient of kinetic friction is non-zero, it will be factored into the theoretical torque computations. Both Coulomb friction and the coefficient of kinetic friction have been integrated into the dynamic calculations.

Regular maintenance and proper lubrication are critical for reducing frictional torque. Although the Coulomb friction of the motor remains relatively stable, it may undergo changes during prolonged operation. Variations in speed can affect viscous friction, thereby altering the frictional torque. Over extended periods, wear on components such as bearings and gears can modify the contact surface conditions, influencing the frictional torque. Additionally, the aging, contamination, or insufficient application of lubricants can increase frictional torque, and the performance differences among various lubricants further contribute to these effects. In this study, static friction torque and kinetic friction coefficients are tabulated and transmitted to the servo system to compensate for the theoretical torque. It is important to note that frictional parameters vary across different joints. Furthermore, both the motor and the robot are susceptible to thermal effects, and incorporating a temperature compensation factor into the theoretical torque calculations is recommended to account for these influences.

Table 4 shows the relationship between the threshold value and the success rate of collision detection, false alarm rate, and missed detection rate.

Owing to the inherent discrepancies between the theoretical torque and the feedback torque, the threshold value must not be established too minimally. With the threshold calibrated at 105%, each collision was promptly identified; however, this configuration was accompanied by a significant false positive rate. Specifically, during ten operational cycles of the robot, collisions were erroneously reported in the complete absence of any physical impact. While the 105% threshold ensured the detection of all collisions, the elevated rate of false positives rendered this threshold inappropriate. Consequently, the threshold was recalibrated to 110%. At this juncture, collisions continued to be detected with alacrity, yet the false positive rate persisted, manifesting as two spurious collision reports over 100 operational cycles devoid of any actual collisions. Upon further adjustment of the threshold to 115%, and after several hundred test cycles without any collisions, no false positives were recorded. Thresholds of 105%, 110%, 115%, and 120% were subjected to empirical testing by introducing an obstacle for the robot to collide with, and all collisions were accurately reported without fail. Thus, it is empirically validated that establishing the threshold for actual collision detection at 120% of the maximum deviation between the theoretical and calculated torque is judicious for practical implementation.

The threshold value is set to 1.2, which is obtained through experiments, and a certain margin is reserved. Because the theoretical torque and feedback torque cannot be completely consistent and synchronized, there are errors between them. Theoretically, if all factors are considered carefully, the error between the theoretical torque and the feedback torque will be very small, so the threshold value should be about 1.05, and the collision can be detected faster. But in the actual operation process, we found through experiments that even if all parameters are verified, there are still errors in the theoretical torque and the calculated torque, especially in the acceleration and deceleration phases, and the errors are more difficult to be adjusted through parameters. If it is found in the experiment that the deviation between the theoretical torque and the actual torque is too large during the normal operation of the robot, a parameter for calculating the theoretical torque may not be accurate, or some factors are not taken into account. It is necessary to adjust the parameters to make the theoretical torque and feedback torque as consistent as possible. But even so, with the increase in running time, the deviation between the theoretical torque and the actual torque will become larger, which is mainly because the feedback torque may be different from the initial running stage with the change in friction, temperature, lubrication, and other factors of the robot. In the experiment, even if the threshold is set to 1.15, all collisions can be successfully detected without false detection. The reason for setting it to 1.2 is that the deviation between theoretical torque and feedback torque will increase due to long-term operation, and the threshold value will be enlarged to 1.2. The advantage of scaling up to 1.2 is that it can avoid false alarms and detect them smoothly, but the disadvantage is that the detection time will be a little slow.

The industrial robot, which includes dynamic collision detection software (Servo v1.10), has undergone MTBF testing at an authoritative third-party testing institution in China and has passed 80,000 h of MTBF testing, proving that the system has high stability.

## 4. Experiment

### 4.1. The Situation During Normal Operation

**Figure 6:**
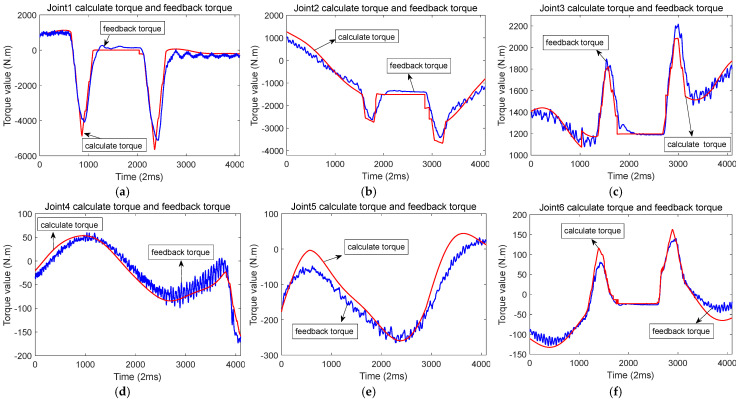
The waveform of calculated torque and feedback torque of each joint during normal operation of the robot. (**a**) Joint 1 calculates the torque and feedback torque; (**b**) Joint 2 calculates the torque and feedback torque; (**c**) Joint 3 calculates the torque and feedback torque; (**d**) Joint 4 calculates the torque and feedback torque; (**e**) Joint 5 calculates the torque and feedback torque; (**f**) Joint 6 calculates the torque and feedback torque.

In the actual testing, a robot with a 50 kg load was used. When there was no collision, i.e., during normal operation, the waveforms of the calculated torque and feedback torque for each axis are shown in Figure 6. The feedback torque is shown by the blue line, while the red line indicates the calculated torque or theoretical torque. The feedback torque and calculated torque of six joints of the robot are sampled in the figure. It is evident that the trajectory of the calculated torque closely matches that of the feedback torque, with the difference between them remaining small. The calculated torque is based on the encoder angle, velocity, and acceleration, whereas the actual torque is derived from motor current sampling. The red line in the figure represents the calculated torque, and the feedback torque is shown by the blue line.

The threshold value is automatically set, and without activating the collision detection function, the robot completes a complete motion once. Find the maximum value Δ of the difference between the calculated torque and the feedback torque throughout the entire motion process. Set 1.2 ×Δ as the threshold value and then activate the collision detection function. This task will be automatically completed by the servo. That is to say, once collision detection is enabled, the first action is to find the collision threshold value. Subsequently, the collision detection function only truly takes effect with the repeated actions of the robot.

### 4.2. X-Axis Collision Experiment

**Figure 7:**
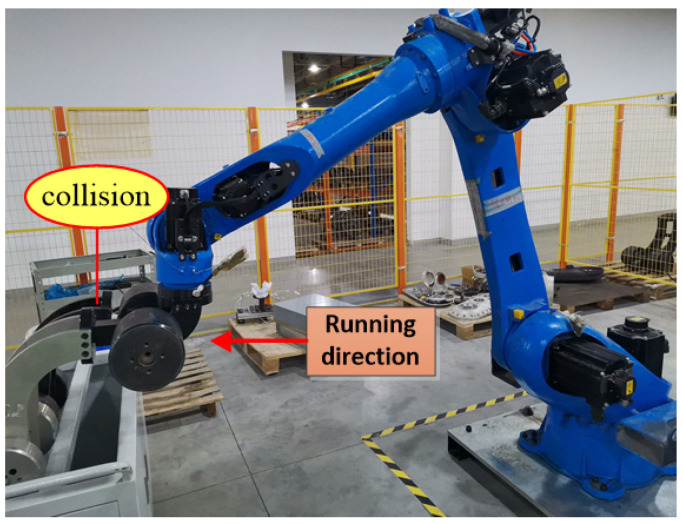
Robots collide while running along the *X*-axis direction.

**Figure 8:**
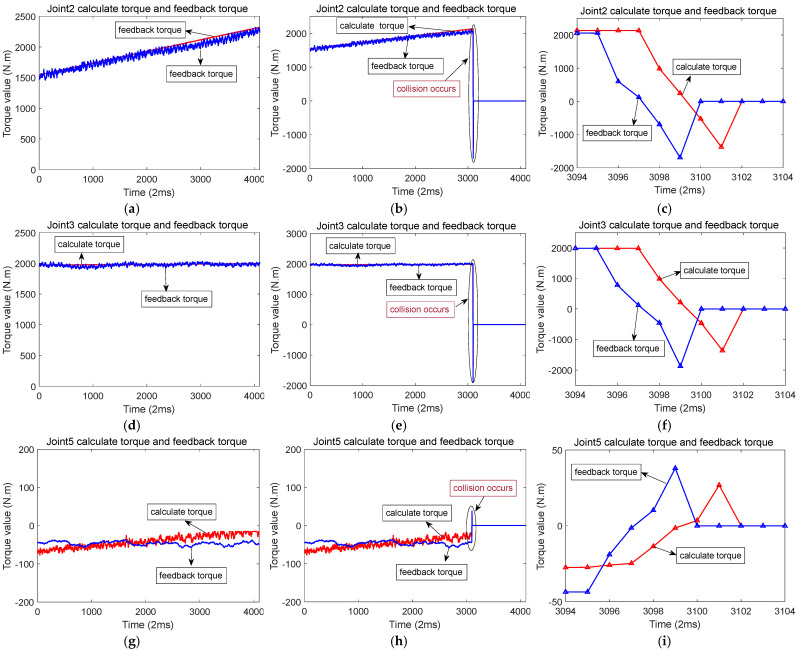
Calculation of torque and feedback torque waveform before and after robot collision in the *X*-axis direction (The red line represents the calculated torque, and the blue line represents the feedback torque). (**a**) When the robot runs along the *X*-axis, the calculated torque and feedback torque of joint 2. (**b**) Calculation of torque and feedback torque of joint 2 before and after the collision. (**c**) When a collision occurs, the calculated torque and feedback torque of joint 2. (**d**) When the robot runs along the *X*-axis, the calculated torque and feedback torque of joint 3. (**e**) Calculation of torque and feedback torque of joint 3 before and after the collision. (**f**) When a collision occurs, the calculated torque and feedback torque of joint 3. (**g**) When the robot runs along the *X*-axis, the calculated torque and feedback torque of joint 5. (**h**) Calculation of torque and feedback torque of joint 5 before and after the collision. (**i**) When a collision occurs, the calculated torque and feedback torque of joint 5.

Figure 7 illustrates the scenario of when a collision occurs along the *X*-axis. While the robot moves along the *X*-axis, joints 2, 3, and 5 are in motion. When a collision occurs in the *X*-axis direction, there is a sudden change in the feedback torque and calculated torque for joints 2, 3, and 5. The changes are particularly pronounced in joints 2 and 3. During a collision, the distinction between the feedback torque and the calculated torque exceeds a predefined threshold, triggering a rapid shutdown to protect the robot from damage. Figure 8a,d,g represents the calculated torque and feedback torque of each joint of the robot during normal operation along the *X*-axis. Figure 8b,e,h represents the moments when the robot collided while running along the *X*-axis, resulting in a sudden change in the calculated torque and feedback torque of each joint. Immediately stop the machine after a collision occurs, so the calculated torque and feedback torque values after the collision are both equal to 0. Figure 8c,f,i shows the enlarged waveforms of the calculated torque and feedback torque for each joint at the moment of collision. In these enlarged sections, the computed torque is shown by the red line, while the feedback torque is shown by the blue line.

### 4.3. Y-Axis Collision Experiment

**Figure 9:**
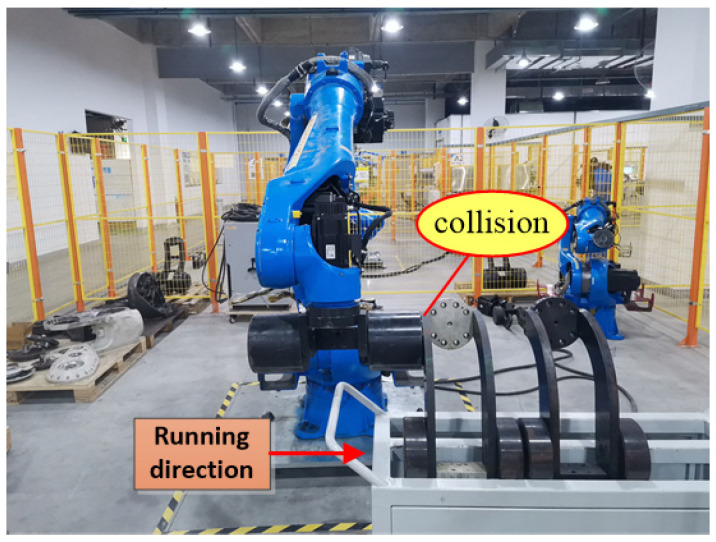
Robots collide while running along the *Y*-axis direction.

Figure 9 shows the scenario of when a collision occurs along the *Y*-axis. During this operation, when the robot moves along the *Y*-axis, joints 1, 2, 3, 5, and 6 all experience motion.

**Figure 10:**
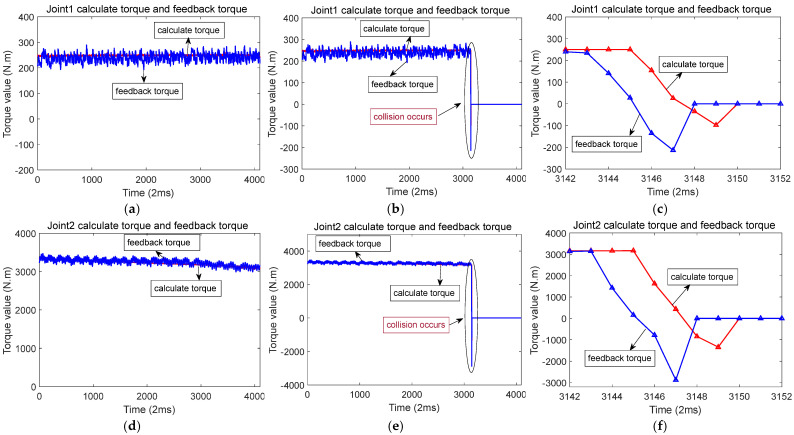

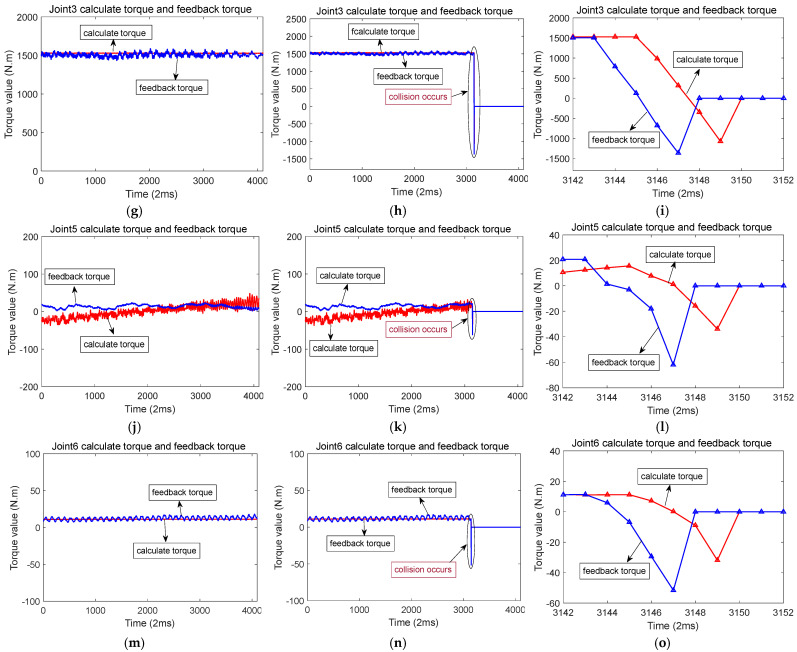
Calculation of torque and feedback torque waveform before and after robot collision in the *Y*-axis direction (The red line represents the calculated torque, and the blue line represents the feedback torque). (**a**) When the robot runs along the *Y*-axis, the calculated torque and feedback torque of joint 1. (**b**) Calculation of torque and feedback torque of joint 1 before and after the collision. (**c**) When a collision occurs, the calculated torque and feedback torque of joint 1. (**d**) When the robot runs along the *Y*-axis, the calculated torque and feedback torque of joint 2. (**e**) Calculation of torque and feedback torque of joint 2 before and after the collision. (**f**) When a collision occurs, the calculated torque and feedback torque of joint 2. (**g**) When the robot runs along the *Y*-axis, the calculated torque and feedback torque of joint 3. (**h**) Calculation of torque and feedback torque of joint 3 before and after the collision. (**i**) When a collision occurs, the calculated torque and feedback torque of joint 3. (**j**) When the robot runs along the *Y*-axis, the calculated torque and feedback torque of joint 5. (**k**) Calculation of torque and feedback torque of joint 5 before and after the collision. (**l**) When a collision occurs, the calculated torque and feedback torque of joint 5. (**m**) When the robot runs along the *Y*-axis, the calculated torque and feedback torque of joint 6. (**n**) Calculation of torque and feedback torque of joint 6 before and after collision. (**o**) When a collision occurs, the calculated torque and feedback torque of joint 6.

Figure 10a,d,g,j,m, shows the calculated torque and feedback torque of each joint during normal operation of the robot along the *Y*-axis. Figure 10b,e,h,k,n show that a collision occurred at a certain moment when the robot was running along the *Y*-axis, resulting in a sudden change in the calculated torque and feedback torque of each joint. Figure 10c,f,i,l,o show the enlarged waveforms of the calculated torque and feedback torque details for each joint at the moment of collision.

### 4.4. Z-Axis Collision Experiment

**Figure 11:**
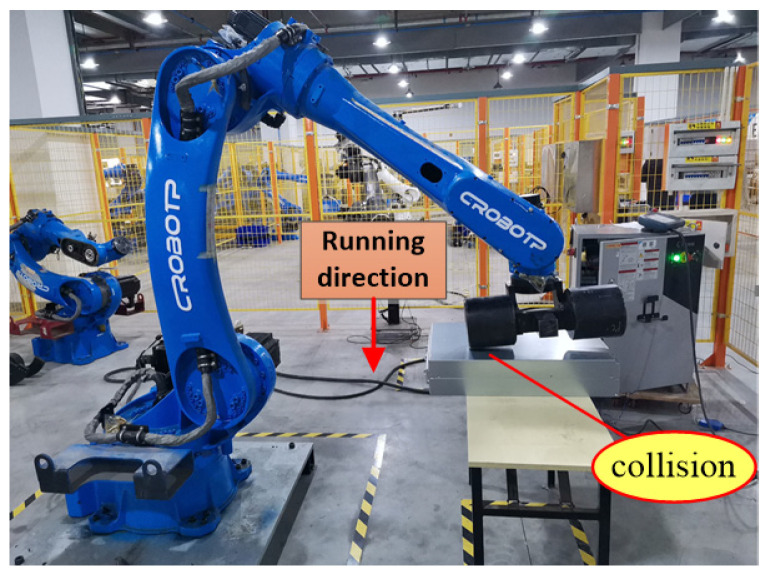
Robots collide while running along the *Z*-axis direction.

When a collision happens along the robot’s *Z*-axis, the torque changes in joint 2 and joint 3 are the most significant. Figure 11 illustrates the scenario of when a collision happens along the *Z*-axis. During this operation, when the robot moves along the *Z*-axis, joints 2, 3, and 5 experience motion.

**Figure 12:**
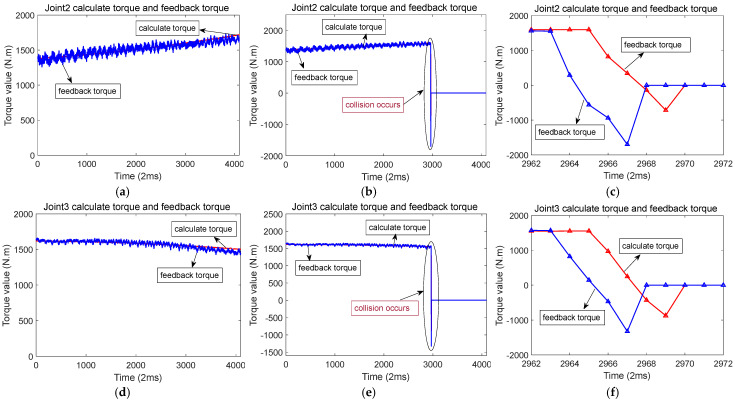

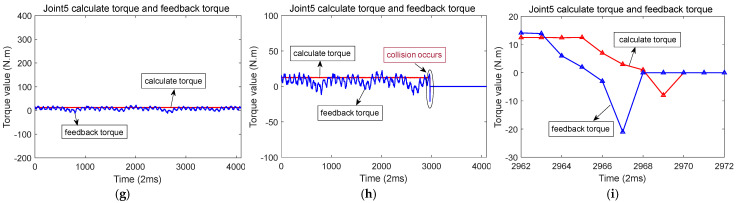
Calculation of torque and feedback torque waveform before and after robot collision in the *Z*-axis direction (The red line represents the calculated torque, and the blue line represents the feedback torque). (**a**) When the robot runs along the *Z*-axis, the calculated torque and feedback torque of joint 2. (**b**) Calculation of torque and feedback torque of joint 2 before and after the collision. (**c**) When a collision occurs, the calculated torque and feedback torque of joint 2. (**d**) When the robot runs along the *Z*-axis, the calculated torque and feedback torque of joint 3. (**e**) Calculation of torque and feedback torque of joint 3 before and after the collision. (**f**) When a collision occurs, the calculated torque and feedback torque of joint 3. (**g**) When the robot runs along the *Z*-axis, the calculated torque and feedback torque of joint 5. (**h**) Calculation of torque and feedback torque of joint 5 before and after the collision. (**i**) When a collision occurs, the calculated torque and feedback torque of joint 5.

Figure 12a,d,g shows the calculated torque and feedback torque during normal operation of the robot along the *Z*-axis. Figure 12b,e,h shows that a collision occurred at a certain moment when the robot was running along the *Z*-axis, resulting in a sudden change in the calculated torque and feedback torque of each joint. Figure 12c,f,i shows enlarged waveforms of the calculated torque and feedback torque details for each joint at the moment of collision.

## 5. Conclusions

This paper utilizes a servo drive dynamic detection module to enable collision detection for industrial robots. Although the dynamic calculations involved are relatively large and time-consuming, collision detection has been successfully implemented in the system, which is crucial for protecting the robot from severe damage. The experimental findings show that the servo drive can precisely and quickly detect a collision through dynamic calculation. When a sudden change in the feedback torque and the deviation from the calculated torque is greater than the set collision threshold value, it can be detected quickly. It can immediately release the brake of the motor and stop the servo from working. This will help protect robots from serious collision injuries. The proposed collision detection solution is low-cost and can, theoretically, handle multiple collisions without rendering the robot inoperable. This approach provides valuable insights for future research. However, further research is required to increase the precision of collision detection based on dynamic calculations and to enhance the speed of collision detection for even quicker response times.

## Figures and Tables

**Table 1:** Collision detection principles and handling methods of some manufacturers.

Robot	Components Used for Collision Detection	Detection Mechanism	Processing Results
UR5(UR)	Dual encoder	Torque/current	Emergency stop
YuMi(ABB)	None	Current	Reverse turn
LBR iiwa(KUKA)	Dual encoder and Torque sensor	Torque	Reverse turn
CR-35iA(FANUC)	Torque sensor	Torque	Emergency stop

**Table 2:** Modified D-H parameter table.

Coordinate System Transformation	Link	$\alpha_{i-1}({}^{\circ})$	$a_{i-1}$/mm	$\theta_{i}({}^{\circ})$	$d_{i}$/m
Base–Link 1	1	0	0	$\theta_{1}(0)$	0
Link 1–Link 2	2	90	320	$\theta_{2}(90)$	0
Link 2–Link 3	3	0	1111	$\theta_{3}(0)$	0
Link 3–Link 4	4	90	205	$\theta_{4}(0)$	0
Link 4–Link 5	5	−90	0	$\theta_{5}(0)$	1232
Link 5–Link 6	6	90	0	$\theta_{6}(0)$	169

**Table 3:** Robot dynamic collision detection parameter file.

Name	Joint 1	Joint 2	Joint 3	Joint 4	Joint 5	Joint 6	Tool
Joint mass (kg)	221.74	66.463	149.58	75.778	19.3	0	50
Centroid coordinate X (m)	−0.14956	−0.58	−0.1364	0.00488	0.001234	0.1	0.25
Centroid coordinate Y (m)	−0.0663	−0.00008	−0.013851	−0.4611	0.0009	0	0.001
Centroid coordinate Z (m)	kg·m^2^	0.1198	0.046264	−0.0514	0.0593	0.18	0.18
$I_{XX}$ (kg·m^2^)	5.0181	0.62859	2.6286	7.2478	0.1718	5	0.01
$I_{YY}$ (kg·m^2^)	10.079	11.092	3.1253	7.0028	0.08182	5	0.01
$I_{ZZ}$ (kg·m^2^)	11.056	0.62859	2.6286	7.2478	0.1718	3	0.01
$I_{XY}$ (kg·m^2^)	0	0	0	0	0	0	0
$I_{YZ}$ (kg·m^2^)	0	0	0	0	0	0	0
$I_{XZ}$ (kg·m^2^)	0	0	0	0	0	0	0
$I_{XX}$ (kg·m^2^)	0	0	0	0	0	0	0
Motor Rated torque (N·m)	26.3	26.3	14.33	4.8	4.8	3.18	0
Motor rated current (A)	24	24	7.6	4.8	4.8	3.5	0
Reduction ratio	210	219.46	145	133.59	157.33	105	1
Coulomb friction force (N·m)	220	574.45	250	40	97.837	33.687	0
Kinetic friction coefficient	530	874.53	250	80	55.84	27.4	0

**Table 4:** The relationship between the threshold value and the success rate of collision detection, false alarm rate, and missed detection rate.

Threshold (%)	Success Rate (%)	False Alarm Rate (%)	Miss Rate (%)
105	100	20	0
110	100	2	0
115	100	0	0
120	100	0	0

## Data Availability

The original contributions presented in the study are included in the article; further inquiries can be directed to the corresponding author.
